# Evaluation of a Tennessee statewide initiative to reduce early elective deliveries using quasi-experimental methods

**DOI:** 10.1186/s12913-019-4033-1

**Published:** 2019-04-02

**Authors:** Michael P. Thompson, Ilana Graetz, Caitlin N. McKillop, Peter H. Grubb, Teresa M. Waters

**Affiliations:** 1 Department of Preventive Medicine, University of Tennessee Health Science Center, 66 N Pauline, Memphis, TN 38163 USA; 2 Department of Cardiac Surgery, University of Michigan Medical School, 5331K Frankel Cardiovascular Center, 1500 E. Medical Center Dr, Ann Arbor, MI 48109 USA; 3 Department of Health Policy and Management, Emory School of Public Health, 1518 Clifton Rd., NE, Suite 636, Atlanta, GA 30322 USA; 4 Department of Economics, SUNY Cortland, Old Main, Room 127, Gerhart Dr., Cortland, NY 13045 USA; 5 Department of Pediatrics, Vanderbilt University School of Medicine, 2200 Children’s Way, Nashville, TN 37212 USA; 6 For the Tennessee Initiative for Perinatal Quality Care (TIPQC) Reducing Early Elective Deliveries Before 39 Weeks EGA Project, 2215B Garland Ave, Nashville, 37232 TN USA; 7 Division of Neonatology, Department of Pediatrics, University of Utah, 295 Chipeta Way, Salt Lake City, UT 84108 USA; 8 Department of Health Management and Policy, University of Kentucky College of Public Health, 111 Washington Avenue, Lexington, KY 40536 USA

**Keywords:** Obstetrics and gynecology, Quality improvement, Evaluation methodology

## Abstract

**Background:**

Concerted quality improvement (QI) efforts have been taken to discourage the practice of early elective deliveries (EEDs), but few studies have robustly examined the impact of directed QI interventions in reducing EED practices. Using quasi-experimental methods, we sought to evaluate the impact of a statewide QI intervention to reduce the practice of EEDs.

**Methods:**

Retrospective cohort study of vital records data (2007 to 2013) for all singleton births occurring ≥36 weeks in 66 Tennessee hospitals grouped into three QI cohorts. We used interrupted-time series to estimate the effect of the QI intervention on the likelihood of an EED birth statewide, and by hospital cohort. We compared the distribution of hospital EED percentages pre- and post-intervention. Lastly, we used multivariable logistic regression to estimate the effect of QI interventions on maternal and infant outcomes.

**Results:**

Implementation of the QI intervention was associated with significant declines in likelihood of EEDs immediately following the intervention (odds ratio, OR = 0.72; *p* < 0.001), but these results varied by hospital cohort. Hospital risk-adjusted EED percentages ranged from 1.6–13.6% in the pre-intervention period, which significantly declined to 2.2–9.6% in the post-intervention period (*p* < 0.001). The QI intervention was also associated with significant reductions in operative vaginal delivery and perineal laceration, and immediate infant ventilation, but increased NICU admissions.

**Conclusions:**

A statewide QI intervention to reduce EEDs was associated with modest but significant declines in EEDs beyond concurrent and national trends, and showed mixed results in related infant and maternal outcomes.

**Electronic supplementary material:**

The online version of this article (10.1186/s12913-019-4033-1) contains supplementary material, which is available to authorized users.

## Background

Early elective deliveries (EEDs), which can be broadly defined as non-medically indicated births occurring during the 37th and 38th week of gestation, are associated with adverse infant and maternal outcomes [1–4]. The practice of EEDs reached its apex in 2008–2009, when they accounted for about 10–15% of all births in the United States [5–9]. In response, several local, regional, and national efforts began focusing on eliminating the practice of EEDs. Consequently, in more recent years, the practice of elective deliveries has declined, with largest declines occurring in early-term births [8, 10, 11].

However, the extent to which local or regional quality improvement (QI) interventions can be credited with declining trends in EEDs has not been robustly examined. To date, several studies have demonstrated that QI interventions, such as those promoting adherence to EED guidelines established by the American College of Obstetricians and Gynecologists (ACOG) or implementing “hard stop” policies, were associated with reductions in the practice of EEDs [6, 12–15]. However, these studies typically relied on simple pre-post analyses to evaluate the effect of the QI intervention to reduce EEDs. These methods do not account for underlying secular trends in clinical practice, which could explain the success of QI interventions. Therefore, a rigorous evaluation using quasi-experimental methods is needed to fully understand the impact of QI interventions on reducing EEDs, independent of concurrent trends.

Beginning in 2009, the State of Tennessee supported pilot programs to reduce the practice of EEDs through a multi-hospital QI intervention, which was ultimately expanded to all birthing hospitals across the state [16, 17]. Leveraging these efforts, we sought to evaluate whether the QI interventions were associated with declines in the likelihood of an EED, over-and-above concurrent trends in EED practices. To accomplish this objective, we used the interrupted-time series method, which is a quasi-experimental analytic method that incorporates concurrent outcome trends in evaluating the effects of policies or QI interventions [18]. Secondarily, we explored whether the QI interventions were associated with changes in the distribution of hospital-level EED percentages. Third, we explored whether the QI interventions were associated with improved infant and maternal outcomes. Finally, we compared temporal trends in EED rates for Tennessee hospitals with those occurring nationwide.

## Methods

### Data and study population

In this retrospective cohort study, we linked birth and death vital record files for all births occurring in the State of Tennessee between 2007 and 2013. The Division of Health Statistics in the Tennessee Department of Health abstracted all birth and death records and created unique identifiers to support file linkage at the birth record level. We also abstracted publically available national birth vital records data from 2007 to 2013 to estimate national EED rates. For Tennessee and national vital records data, we excluded births if they were non-singleton births, had an estimated gestational age less than 37 weeks, or occurred in non-hospital settings.

### Intervention

The primary independent variable in our analyses was the announcement of the EED reduction QI interventions, which occurred at different dates for different hospitals across the state. A description of the three hospital cohorts (*N* = 66 total hospitals), including the pre- and post-intervention dates, sample sizes, and primary intervention approaches can be found in Additional file 1. Briefly, cohorts 1 and 2 participated in a pilot multi-center QI project sponsored by the Tennessee Initiative for Perinatal Quality Care (TIPQC), Tennessee’s statewide perinatal QI collaborative [19]. In this first maternal project, TIPQC applied a modified Breakthrough Collaborative approach to encourage data-driven implementation of process changes, while also addressing participant concerns about sharing identifiable data [20]. Cohort 1 hospitals (*N* = 5) initiated their pilot program in April 2009, and submitted patient-level reason-for-delivery data, and received quarterly feedback on aggregate data through local leader conference calls. Cohort 2 hospitals (*N* = 4) joined Cohort 1 hospitals in April 2010, which modified the initial pilot program with added on-demand reports of local QI data and monthly webinars to review aggregate data and share lessons learned during implementation. Cohort 3 hospitals (*N* = 57) joined in April 2012 and was sponsored jointly by TIPQC and the Tennessee Hospital Association and was aligned with Medicare’s Partnership for Patients Hospital Engagement Network initiative to reduce EEDs. Cohort 3 interventions included a “hard stop” policy, monthly feedback of Joint Commission Perinatal Core 5 EED rates and sharing cohort 1 and 2 EED reduction experience during monthly webinars adopted from cohort 2 [17]. A more thorough description of TIPQC and the EED QI project can be found in Additional file 2.

### Outcomes

The primary outcome in this study was EED birth status (yes vs. no). Early-term births were those births occurring between 37 0/7 weeks and 38 6/7 weeks of gestation, whereas full-term births were those occurring at 39 weeks and greater [9, 21]. Since vital records do not have data on elective birth status, we categorized elective birth status based on the presence of medical indications as defined by ACOG, including previous cesarean section deliveries, pre-pregnancy or gestational hypertension, pre-pregnancy or gestational diabetes, small for gestational age (birthweight < 2500 g), chorioamnionitis, or premature rupture of membranes [1]. We then calculated the percent of births considered to be EEDs using the fetuses-at-risk method, where the denominator is all births with the potential for an EED, i.e., all births 37 0/7 weeks or greater [22].

We also examined several maternal and infant outcomes that might be affected by changes in EED practice. Maternal outcomes included operative vaginal delivery (forceps or vacuum), perineal laceration, prolonged labor, blood transfusion, and unplanned operation or hysterectomy. Infant outcomes included: Apgar < 7 at 5 min, immediate ventilation, antibiotic administration, NICU admission, neonatal mortality (within 28 days), and infant mortality (within 1 year).

### Covariates

Maternal covariates for adjustment in multivariable models were also abstracted from vital records data, and included maternal age (in years), race (white vs. black or other), Hispanic ethnicity (yes vs. no), more than a high school education (yes vs. no), insurance type (private vs. Medicaid or other), annual income (<$25,000 vs $25,000+), prenatal visits (less than five vs. five or more), and nulliparous vs. multiparous. We compared these characteristics by pre- and post-intervention status using chi-square tests and ANOVA for categorical and continuous variables, respectively.

### Statistical analysis

We used an interrupted-time series (ITS) approach to estimate the effect of the QI interventions on the likelihood of an EED birth. This quasi-experimental method is commonly used to estimate the impact of an intervention or policy on health outcomes, particularly when no clear control group exists [18]. By segmenting temporal trends into pre- and post-intervention periods, we can examine how the intervention affected the overall event percentage (model intercept) and change in event percentage over time (model slope). Our primary analysis used a hierarchical logistic regression model, which specified as logit [EED = 1] = β_0_ + β_1_*quarter + β_2_*intervention + β_3_*quarter*intervention + βX + b_0j_ + ε. In this model, quarter is the continuous quarterly time variable, intervention is a categorical dummy variable indicating whether the birth occurred in the pre- or post-announcement of the QI initiatives, βX is a set of maternal covariates and coefficients, b_0j_ represents the random effect for hospital *j*, and ε represents the model error. From this model, we estimated the pre-intervention quarterly trend (β_1_), the change in overall event percentage at implementation (β_2_), the post-intervention quarterly trend (β_1_ + β_3_), and the change in EED event percentage over time (β_3_). We performed this analysis in the overall statewide sample, adjusting for cohort, maternal covariates, and hospital random effects. Because the interventions were not rolled out simultaneously, we also repeated the analysis by cohort, adjusting for maternal covariates and hospital random effects only.

We estimated hospital-level risk-adjusted EED percentages using the hierarchical logistic regression model, which estimates the hospital-level deviation in adjusted EEDs compared to the average hospital, after adjusting for maternal characteristics. We created box plots of the adjusted EED percentages for the pre- and post-intervention periods for the overall statewide sample, and by hospital cohort. We used ANOVA to test for significant differences in the distribution of hospital-level risk adjusted EED percentages before and after the QI interventions.

We also explored whether the interventions were associated with changes in maternal and infant outcomes. Because most of these outcomes rarely occurred, standard logistic regression models were used to examine changes in outcomes. Models were adjusted for the same maternal covariates listed above and hospital cohort.

Lastly, to place our findings in the context of overall national trends, we estimated quarterly EED rates by Tennessee hospital cohort and at the national level. We overlaid fitted Loess curves for the quarterly rates in the Tennessee hospital cohorts and at the national level for visual comparison.

This study was deemed non-human subject research because it made use of retrospective and de-identified data. All analyses were conducted using SAS version 9.4 (SAS Institute Inc., Cary, NC).

## Results

After exclusions, we identified 149,333 births occurring in the pre-intervention period(s), and 133,840 births occurring in the post-intervention period(s) (Table 1). In total, there were 58,175 births (20.5%) in five cohort 1 hospitals, 41,579 births (14.7%) in four cohort 2 hospitals, and 183,419 births (64.8%) in the remaining 57 cohort 3 hospitals. Maternal characteristics in pre- and post-intervention periods are also presented in Table 1. Briefly, in the post-intervention period(s) there were more births involving older mothers who were more likely to be non-white, and have more than a high school education, have private insurance, and be nulliparous.

**Table 1:** Maternal characteristics for sample by intervention status

*Maternal Characteristic*	*Overall* *(n = 283,173)*	*Intervention Period*		*P-value*
		*Pre* *(n = 149,333)*	*Post* *(n = 133,840)*	
Age, mean ± SD	26.6 ± 5.8	26.4 ± 5.9	26.7 ± 5.8	< 0.0001
Race, n (%)				0.0379
*White*	189,534 (66.9)	99,708 (66.8)	89,826 (67.1)	
*Black*	60,947 (21.5)	32,179 (21.6)	28,768 (21.5)	
*Other*	32,692 (11.5)	17,446 (11.7)	15,246 (11.4)	
Hispanic Ethnicity, n (%)	27,004 (9.5)	14,459 (9.7)	12,545 (9.4)	0.0052
More than a High School Education, n (%)	147,846 (52.2)	76,326 (51.1)	71,520 (53.4)	< 0.0001
Insurance Type, n (%)				< 0.0001
*Private*	143,306 (50.6)	74,704 (50.0)	68,602 (51.3)	
*Medicaid*	112,887 (39.9)	59,867 (40.1)	53,020 (39.6)	
*Other*	26,980 (9.5)	14,762 (9.9)	12,218 (9.1)	
Annual Income <$25,000, n (%)	105,231 (37.2)	54,973 (36.8)	50,258 (37.6)	< 0.0001
Prenatal visits ≥5, n (%)	246,204 (86.9)	129,815 (86.9)	116,389 (87.0)	0.8043
Nulliparous, n (%)	117,108 (41.4)	62,327 (41.7)	54,781 (40.9)	< 0.0001

Abbreviations: *SD* standard deviation

**Fig. 1:**
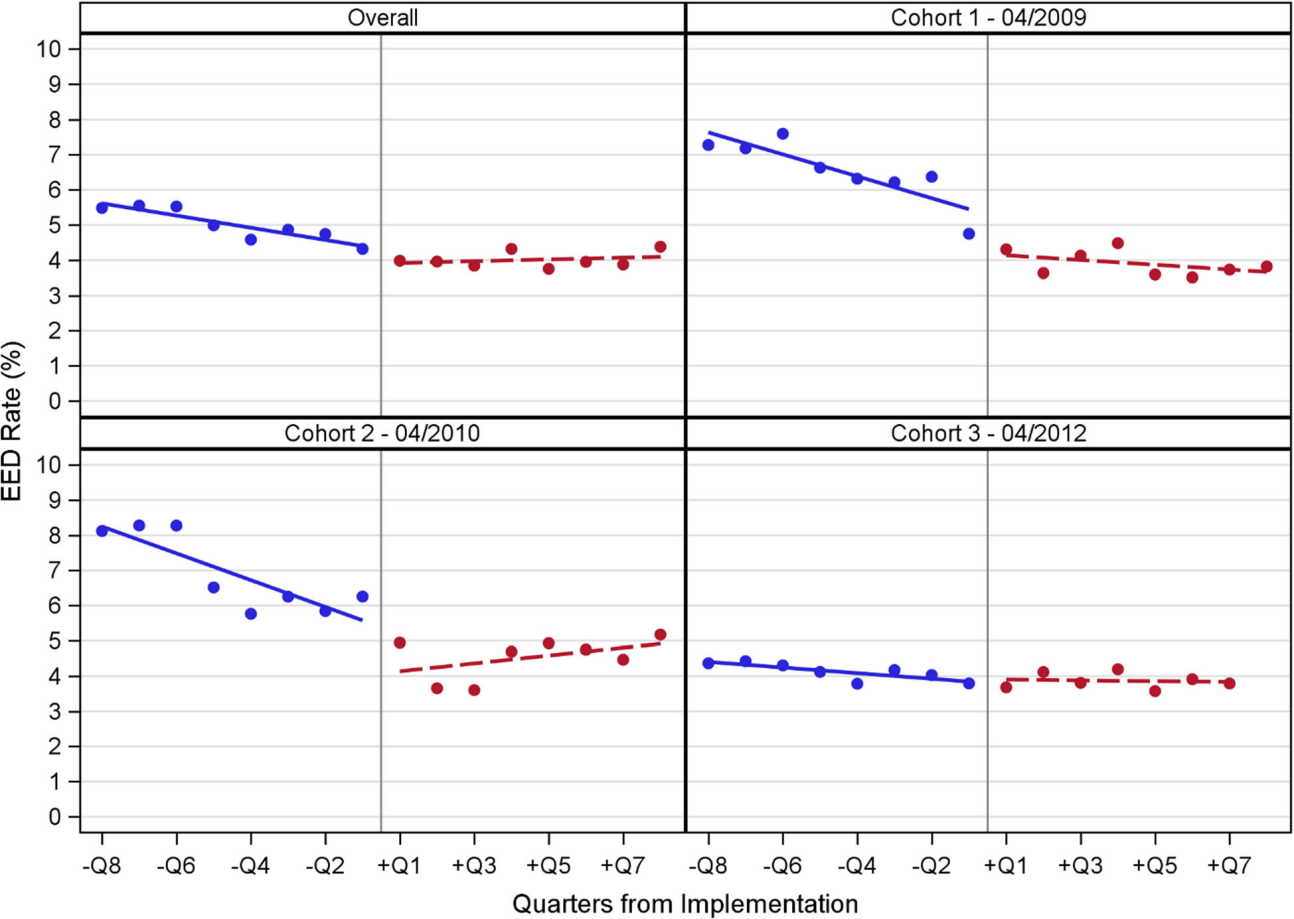
Quarterly EED percentages during the pre- (blue) and post-intervention (red) for the overall sample, and by cohort

Fig. 1 shows quarterly EED percentages for the overall sample and by cohort, with separate fitted regression lines for the pre- and post-intervention periods. For the overall sample, the baseline pre-intervention EED percentage was approximately 6%, which declined to about 4% in the post-intervention period. Initial EED percentages were higher for cohorts 1 and 2 (8%), compared to cohort 3 (4–5%). By the end of the post-intervention period, EED percentages declined to 4% in cohort 1, 5% in cohort 2, and 4% in cohort 3.

The results of the ITS analysis are displayed in Table 2. For the overall statewide sample, the likelihood of an EED birth was significantly declining during both pre- and post-intervention periods. Overall, the intervention was associated with a significant decline in the probability of EEDs (OR = 0.72; *p* = 0.0002). When we stratified by hospital cohort, we found statistically significant associations between the QI intervention and the likelihood of an EED birth for cohort 1 (OR = 0.57; *p* = 0.0012) and cohort 2 (OR = 0.36; *p* < 0.0001), but not cohort 3 (OR = 0.88; *p* = 0.2655). The change in quarterly trend was attenuated for the overall sample (OR = 1.03, p = 0.0012), cohort 1 (OR = 1.03; p = 0.0012), and cohort 2 (OR = 1.03; *p* = 0.0439), but not for cohort 3 (OR = 1.02, *p* = 0.1613).

**Table 2:** Interrupted-time series analysis of intervention to reduce EEDs for overall sample and by cohort

	*Pre-Intervention* *Quarterly Trend*		*Intervention Change*		*Post-Intervention* *Quarterly Trend*		*Change in Quarterly Trend*	
	*Adjusted Odds Ratio* ^*a*^ *(95% CI)*	*P-value*	*Adjusted Odds Ratio* ^*a*^ *(95% CI)*	*P-value*	*Adjusted Odds Ratio* ^*a*^ *(95% CI)*	*P-value*	*Adjusted Odds Ratio* ^*a*^ *(95% CI)*	*P-value*
Overall	0.97 (0.96–0.98)	< 0.0001	0.72 (0.61–0.86)	0.0002	0.99 (0.98–1.07)	0.3679	1.03 (1.01–1.04)	0.0012
Cohort 1	0.95 (0.93–0.97)	< 0.0001	0.57 (0.40–0.80)	0.0012	0.99 (0.96–1.01)	0.3070	1.03 (1.00–1.07)	0.0439
Cohort 2	0.94 (0.92–0.97)	< 0.0001	0.36 (0.24–0.53)	< 0.0001	1.02 (0.99–1.05)	0.1198	1.09 (1.05–1.13)	< 0.0001
Cohort 3	0.98 (0.97–1.00)	0.0136	0.88 (0.70–1.10)	0.2655	1.00 (0.98–1.02)	0.8783	1.02 (0.99–1.04)	0.1613

^a^Models are adjusted for maternal age (in years), race (white vs. black or other), Hispanic ethnicity (yes vs. no), less than high school education (yes vs. no), insurance type (private vs. Medicaid or other), annual income <$25,000 vs $25,000+, less than five vs. five or more prenatal visits, and nulliparous vs. multiparous

**Fig. 2:**
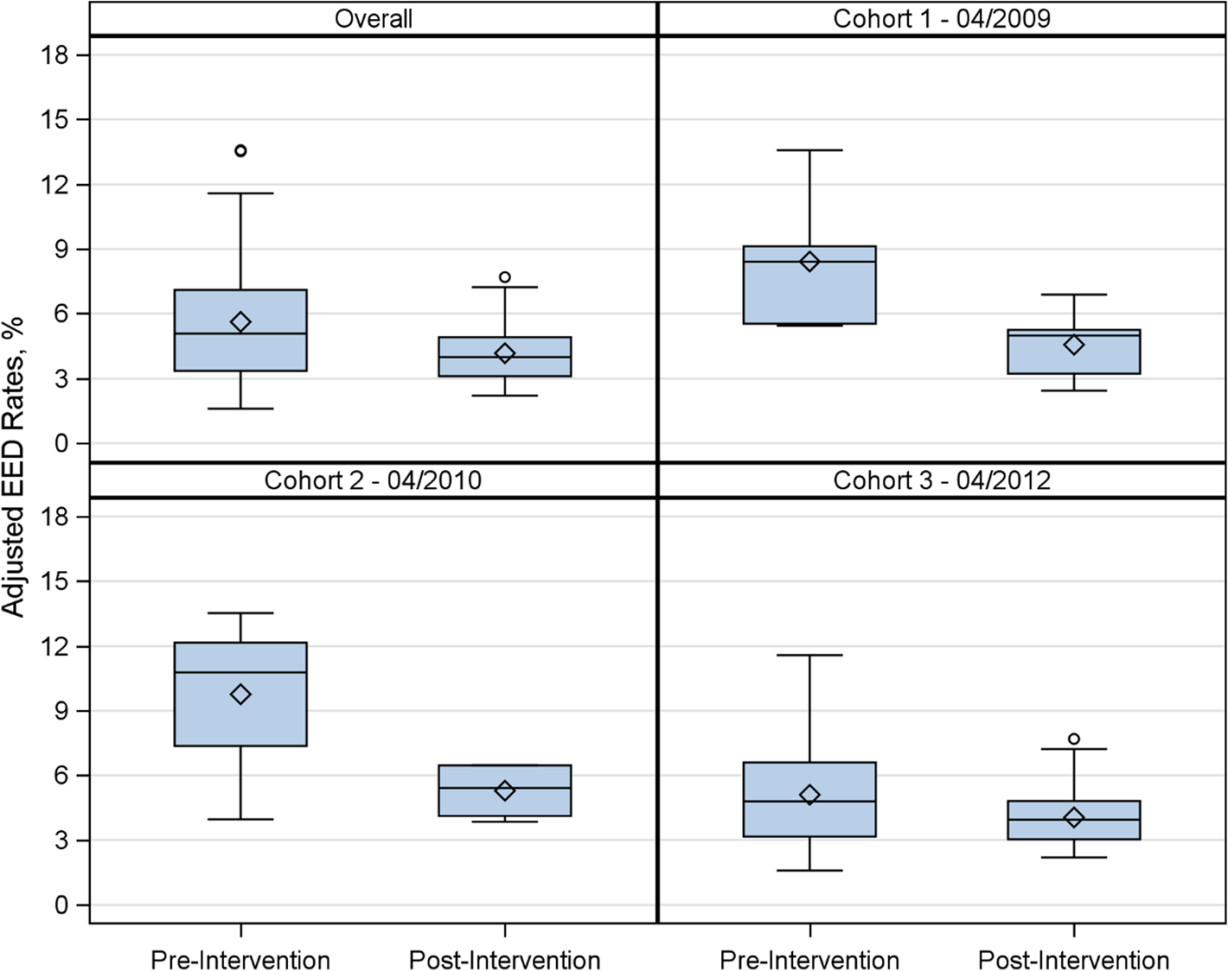
Box plots of adjusted hospital-level EED percentages pre- and post-intervention for the overall sample, and by cohort

The box plots in Fig. 2 illustrate the distribution of hospital-level adjusted EED percentages for the pre- and post-intervention periods. In the overall statewide sample, hospital-level adjusted EED percentages ranged from 1.6 to 13.6% in the pre-intervention period, and ranged from 2.2 to 7.7% in the post-intervention period (*p* < 0.0001). This finding was similar when we stratified by cohort (all *p* < 0.05).

Unadjusted maternal and infant outcome proportions (per 1000 births) and adjusted odds ratios comparing pre- and post-intervention periods are displayed in Table 3. After adjustments, the QI interventions were associated with fewer operative vaginal deliveries (OR = 0.86; *p* < 0.0001) and perineal lacerations (OR = 0.91; *p* = 0.0216), from pre- to post-intervention periods. We also found that the QI interventions were associated with fewer infants needing immediate ventilation (OR = 0.74; p < 0.0001), but an increased likelihood of NICU admissions (OR = 1.10; p < 0.0001).

**Table 3:** Unadjusted and adjusted changes in maternal and infant outcomes pre- and post-intervention

*Outcomes*	*Unadjusted Outcomes per 1000 Births*			*Adjusted* *Odds Ratio* ^*b*^ *(95% CI)*	*P-value*
	*Pre-Intervention* *(n = 149,333)*	*Post-Intervention* *(n = 133,840)*	*Crude Change*		
Maternal					
Operative Vaginal Delivery^a^	69.1	60.8	− 8.3	0.86 (0.83–0.89)	< 0.0001
Perineal Laceration^a^	13.7	12.6	− 0.9	0.91 (0.84–0.99)	0.0216
Prolonged Labor	6.3	5.8	−0.5	0.92 (0.84–1.02)	0.1080
Blood Transfusion	1.9	1.8	−0.1	0.99 (0.83–1.17)	0.8816
Unplanned Operation or Hysterectomy	1.4	1.3	−0.1	0.90 (0.74–1.11)	0.3242
Infant					
Apgar < 7 at 5 min	20.5	20.5	0.0	1.01 (0.96–1.07)	0.5827
Ventilation	49.6	37.9	−11.7	0.74 (0.71–0.77)	< 0.0001
Antibiotics	17.9	17.2	−0.7	0.95 (0.90–1.01)	0.1011
NICU Admission	54.1	59.2	+ 5.1	1.10 (1.07–1.14)	< 0.0001
Neonatal Mortality	4.9	4.8	−0.1	1.03 (0.92–1.14)	0.6189
Infant Mortality	6.7	6.7	0.0	1.04 (0.95–1.14)	0.4406

^a^Excludes cesarean births

^b^Models are adjusted for maternal age (in years), race (white vs. black or other), Hispanic ethnicity (yes vs. no), less than high school education (yes vs. no), insurance type (private vs. Medicaid or other), annual income <$25,000 vs $25,000+, less than five vs. five or more prenatal visits, and nulliparous vs. multiparous

The comparison of Tennessee and national quarterly trends in EEDs from 2007 through 2013 can be seen in Additional file 3. The vertical lines represent the intervention dates for the three hospital cohorts. Compared to national rates, Tennessee hospitals typically had higher EED rates prior to 2009, and declined precipitously to below the national average in all cohorts by 2010, where they remained through the end of 2013.

## Discussion

Through the application of quasi-experimental methods to vital records data, we found that statewide QI interventions in Tennessee hospitals were associated with modest but significant reductions in the likelihood of an EED birth. These reductions were driven by improvements achieved in the first two hospital cohorts (i.e. cohorts 1 and 2). We also found that these improvements were sustained for the two years following the intervention. While we found no significant effect of the intervention in the average EED percentages for cohort 3, pre-intervention EED percentages had already declined substantially prior to the intervention date. Finally, in all three cohorts, we found that the intervention was associated with significant declines in between-hospital variation in EED percentages. This suggests that hospital-level variation in the practice of EEDs were more consistent after implementation of the intervention.

Many studies have demonstrated reductions in EEDs using a variety of intervention approaches, but most of these studies use pre-post, post trend, or statistical process control methods to evaluate their interventions [6, 12–15]. These methods do not account for underlying trends that occur in the background. In such cases, it becomes difficult to disentangle the effect of an intervention from other trends and forces that are occurring in the background that are unrelated to the intervention being studied. For instance, since 2008, studies have reported steadily declining trends in rate of EEDs across the country [8, 9]. In this study, we attempted to control for reported underlying secular trends in EEDs by using ITS methods, which are particularly useful when randomization is not possible, or when evaluation occurs retrospectively [18]. We found that the QI interventions did have a significant effect on the practice of EEDs, but that improvements were largely successful in earlier cohorts. Without adjusting for secular trends, we may incorrectly attribute reductions in EEDs to the QI intervention.

Fewer studies have examined the extent to which interventions reduced hospital-level variation in EED practices over time [14]. Reducing variation in clinical processes is a critical component of quality improvement, as it indicates providers are reducing heterogeneity in clinical practice, and are converging on an accepted clinical practice [23]. Even in cohort 3, where no measureable impact of the intervention on the EED was discernable, the variation in EED percentages across hospitals was significantly reduced after the QI intervention. This finding suggests that measureable improvements in quality were being achieved through reduction in outlier hospitals with high baseline EED percentages, even if no improvements in the aggregate EED percentages were observed.

While national EED trends declined steadily over time, reductions in Tennessee occurred precipitously between 2008 and 2010, with all cohorts moving from above to below the national trend. Reductions in cohorts 1 and 2 aligned closely with the interventions, with some reductions preceding the intervention. Conversely, reductions in cohort 3 occurred well before the intervention began in 2012. The pre-intervention reductions observed in cohort 3 hospitals could reflect a “rising tide” phenomenon, whereby sites not participating in interventions will improve independently or in response to heightened awareness [24]. It is also possible that reductions in EEDs could be attributed to earlier hospital system and national efforts to reduce the practice of EEDs in response to earlier ACOG guidelines [14, 25]. While these and similar efforts likely contributed to early improvements, neither Tennessee nor national EED trends dropped precipitously following the release of the 2009 national guidelines, suggesting that the dramatic reductions in EEDs associated with cohort 1 and 2 implementation may have influenced practice in cohort 3 hospitals prior to its formal start date.

Our study also included investigation of infant and maternal outcomes, which represent important areas of focus, and are not often included in other evaluative studies. Following QI implementation, we found evidence of substantial declines in adverse outcomes, including operative vaginal deliveries, perineal laceration, prolonged labor, and infant ventilation. As these adverse outcomes have been previously linked to EEDs, declining this practice could be an underlying factor for a reduction in adverse outcomes [26] However, other important outcomes such as neonatal ICU admissions rose during the post-intervention period as well. There has been a recently documented rise in NICU admissions more generally, which may contribute to this observation [27]. Ultimately, closer examination of declines in EEDs and changes in adverse outcomes with more clinically robust data is needed to fully understand the impact of this QI initiative on clinical outcomes.

There are limitations to our study. First, vital records data are not research databases and can be limited in their ability to identify delivery events and risk factors [28–31]. Replication of our findings with more clinically relevant data may help identify the extent to which patient factors contributed to differences in EED changes across hospitals. Moreover, our evaluation did not include a qualitative exploration of the local cultural, leadership, or policy changes that might have contributed to declines in EEDs. Second, we assumed that all births that were not medically indicated were elective. Although the criteria we used to define non-medically indicated have been previously published and applied in other studies [1], some births may be misclassified. Third, our study did not randomize hospitals into the study cohorts, and no true control group existed. In fact, our data suggest that many hospitals may have begun reducing EEDs prior to announcement of their participation, as evidenced by low pre-intervention EED rates in cohort 3. However, by using ITS methodologies, we were able to ameliorate some of the bias typically found in observational studies. Nevertheless, confounding by factors not included in our study may still bias our results. Fourth, we were unable to apply the ITS method to maternal and infant outcomes. Thus, underlying trends may explain some of these findings.

## Conclusions

To conclude, QI interventions to reduce the practice of EEDs in the State of Tennessee were associated with modest but significant reductions in the likelihood of EED births and hospital-level variation in EEDs percentages. However, the effect of the QI intervention varied by hospital cohort, and was strongest in the cohorts initiating QI interventions earliest. Finally, we found that the QI intervention had mixed results on infant and maternal outcomes. Future work should clarify the link between EED reduction initiatives and maternal and infant outcomes.

## Additional files

Additional file 1: **Table S1**. Cohort pre- and post-intervention dates, sample sizes, and intervention description. (DOCX 13 kb)

Additional file 2: Appendix. Description of Tennessee Initiative for Perinatal Quality Care (TIPQC). (DOCX 14 kb)

Additional file 3: **Figure S1.** Fitted Loess curves for quarterly EED percentages for Tennessee hospital cohorts compared to national trends (note: vertical lines represent intervention dates for cohorts 1, 2 and 3, respectively). (DOCX 110 kb)

## Abbreviations

ACOG: American College of Obstetricians and Gynecologists
EED: Early Elective Delivery
ITS: Interrupted Time Series
QI: Quality Improvement
TIPQC: Tennessee Initiative for Perinatal Quality Care
