# Supplementary material for: Evaluation of a Tennessee statewide initiative to reduce early elective deliveries using quasi-experimental methods
Source: BMC Health Serv Res. 2019 Apr 2;19:208. doi: 10.1186/s12913-019-4033-1 (PMC6444673; doi:10.1186/s12913-019-4033-1)
Supplement: Supplementary file 1 — Table S1. Cohort pre- and post-intervention dates, sample sizes, and intervention description. (DOCX 13 kb) [file 12913_2019_4033_MOESM1_ESM.docx]

| **Supplemental Table.** Cohort pre- and post-intervention dates, sample sizes, and intervention description. | | | | | | | |
| --- | --- | --- | --- | --- | --- | --- | --- |
|  | *Hospitals, N* | *Total Births, n (%)* | *Pre-Intervention* | | *Post-Intervention* | | *Intervention Description* |
|  |  |  | *Dates* | *Births, n (%)* | *Dates* | *Births, n (%)* |  |
| Overall | 66 | 283,173 (100) | - | 149,333 (100) | - | 133,840 (100) | - |
| Cohort 1 | 5 | 58,175 (20.5) | 4/07 – 3/09 | 29,430 (19.7) | 4/09 – 3/11 | 28,745 (21.5) | Quality improvement collaborative (QIC) model, Patient-level data on reason for delivery, quarterly review of data |
| Cohort 2 | 4 | 41,579 (14.7) | 4/08 – 3/10 | 21,684 (14.5) | 4/10 – 3/12 | 19,895 (14.9) | Cohort 1 intervention plus: cohort 1 mentors cohort 2, on-demand local QI data reports, monthly QI webinar with review of aggregate data |
| Cohort 3* | 57 | 183,419 (64.8) | 4/10 – 3/12 | 98,219 (65.8) | 4/12 – 12/13 | 85,200 (63.7) | “Hard Stop” policy, Hospital Engagement Network alignment, Monthly hospital PC-05 rate data, monthly feedback on hospital rate. |
| * Since data were limited to 2013, the post-intervention period for cohort 3 is 21 months, rather than 24 months. | | | | | | | |
