# Supplementary material for: Evaluation of a Tennessee statewide initiative to reduce early elective deliveries using quasi-experimental methods
Source: BMC Health Serv Res. 2019 Apr 2;19:208. doi: 10.1186/s12913-019-4033-1 (PMC6444673; doi:10.1186/s12913-019-4033-1)
Supplement: Supplementary file 2 — Appendix. Description of Tennessee Initiative for Perinatal Quality Care (TIPQC). (DOCX 14 kb) [file 12913_2019_4033_MOESM2_ESM.docx]

**APPENDIX**

**Description of Tennessee Initiative for Perinatal Quality Care (TIPQC)**

All information regarding the TIPQC and its quality improvement projects referenced below, including the EED Before 39 Weeks Project, can be found on its webpage: TIPQC.org. The TIPQC seeks to improve health outcomes for mothers and infants in Tennessee by engaging key stakeholders in a perinatal quality collaborative that will identify opportunities to optimize maternal and infant outcomes and implement data-driven provider- and community-based performance improvement initiatives. Its goal is to 1.) establish a statewide repository of perinatal data for quality improvement initiatives, 2.) foster state-wide quality improvement initiatives to lower infant & maternal mortality and morbidity and improve outcomes, and 3.) promote system changes by provider organizations to increase use of evidence based clinical practices for newborns, infants, prenatal & postnatal patients & families.

**TIPQC Early Elective Deliveries Before 39 Weeks Project**

**Project Aim:** Using a collaborative quality improvement approach, this project seeks to report the rate and indications for scheduled deliveries before 39 weeks of gestation at the time of scheduling for all scheduled deliveries in Tennessee.

In 2009, participants at the first TIPQC Annual Meeting voted to begin a multi-phase project to address excessive early elective deliveries in Tennessee. The project was completed in 3 phases. A small-scale pilot project was completed in Davidson county in 2009-2010. All Davidson county hospitals with delivery services participated and demonstrated a reduction in their early elective delivery rate prior to 39 weeks gestation. Subsequently hospitals in Hamilton, Jackson, Maury and Shelby counties were asked to join the Davidson county hospitals to test the spreadability of the project. The Davidson county hospitals were able to sustain their reduction in early elective deliveries and the new hospitals demonstrated they were able to achieve similar reductions by the close of 2011.

Statewide spread to the remaining hospitals in Tennessee, through The 39 Week Elective Delivery Reduction began in May 2012 in collaboration with the Tennessee Hospital Association’s (THA) Hospital Engagement Network (HEN) using the Joint Commission’s Perinatal Core Measure 1. In October 2012, a letter from the Tennessee Department of Health (TDH), THA, March of Dimes (MOD), and TIPQC was sent to all hospitals CEOs encouraging adoption of a “hard stop” policy to eliminate Early Elective Deliveries, and included a public commitment pledge, which 54 hospitals signed. The “Healthy Babies Are Worth the Wait” public awareness Campaign sponsored by the MOD, THA, TDH and TIPQC began in November 2012 with a goal of helping the public better distinguish medically necessary from elective deliveries prior to 39 weeks completed gestation. Sixty-six delivery hospitals in Tennessee hospitals have participated in the joint TIPQC, THA, TDH, MOD. The active QI interventions in this project concluded in 2015, and is currently in sustainment.
