# Supplementary material for: Evaluation of a Tennessee statewide initiative to reduce early elective deliveries using quasi-experimental methods
Source: BMC Health Serv Res. 2019 Apr 2;19:208. doi: 10.1186/s12913-019-4033-1 (PMC6444673; doi:10.1186/s12913-019-4033-1)
Supplement: Supplementary file 3 — Figure S1. Fitted Loess curves for quarterly EED percentages for Tennessee hospital cohorts compared to national trends (note: vertical lines represent intervention dates for cohorts 1, 2 and 3, respectively). (DOCX 110 kb) [file 12913_2019_4033_MOESM3_ESM.docx]

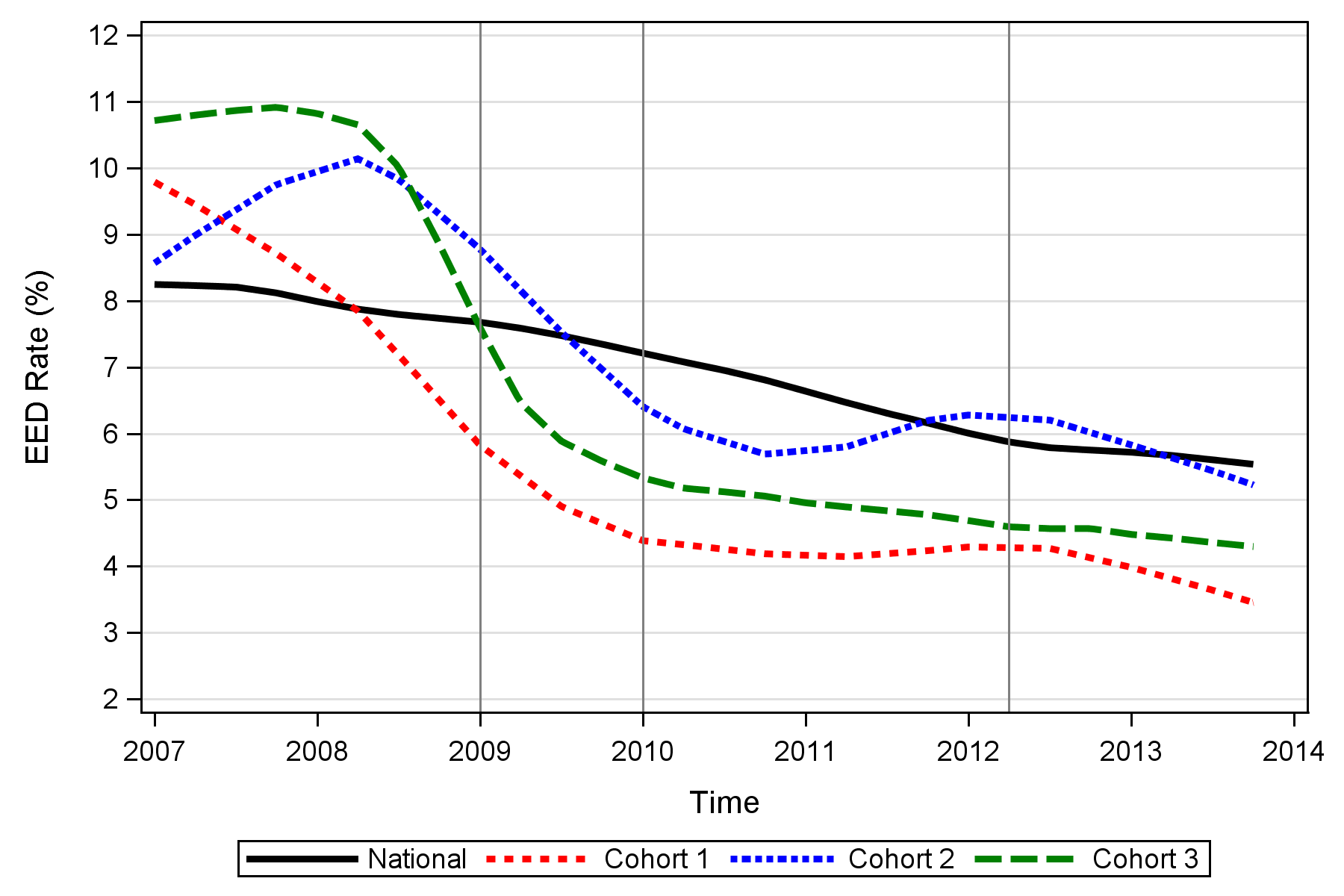


**Supplemental Figure.** Fitted Loess curves for quarterly EED percentages for Tennessee hospital cohorts compared to national trends (note: vertical lines represent intervention dates for cohorts 1, 2 and 3, respectively).
